# Dr. Knox on Amaurosis

**Published:** 1830-07-01

**Authors:** 


XXIII.

ON AMAUROSIS. By JAMES J. KNOX, M. D. &c.

IN the tenth number of our highly respected Glasgow contemporary, for May of the present year, Dr. Knox has published a very valuable and practical paper on amaurosis, and promises to favour his brethren with a paper in continuation. It is one of those communications (somewhat rare indeed) which we cannot much abridge without great detriment to the author. In giving it a wider circulation than it otherwise would have, we shall be doing no injury to the writer, and some benefit to our readers.

Recent investigations have proved that amaurosis depends on an affection either of the optic nerve or the retina, though pathological anatomy is not always able to explain the nature of the lesion. The affection comes on sometimes gradually, sometimes very suddenly—vision being occasionally lost in a single day or night. The symptoms, though very numerous, and sometimes rather doubtful, may be conveniently divided into those felt by the patient, and those seen by the practitioner.

"Although amaurosis does not uniformly commence with the same symptoms, still a troublesome sensation of vivid balls of fire shooting across the eye, and of an innumerable host of mots or black stars floating before it and intercepting vision, are amongst the first warnings which a patient about to be attacked with amaurosis receives. These bodies are sometimes fixed, and at others constantly floating before the eye; they are also of various colours, being at one time black, or green, and occasionally of a fine purple hue.

"The amaurotic patient invariably refers his blindness to the existence of these bodies, and hence it is, that they are so remarkably minute in their description of them. In fact, there is scarcely any thing, however ridiculous, with which at times they are not compared. At present, there is a patient under my care, who complains of vision being intercepted by a large greenish body constantly floating before the eye, and compared by her to a wave of the sea. In another patient, a black body resembling a finger stretches across the eye. They are also frequently compared to birds, insects, &c. It also sometimes happens that these moveable bodies unite, forming a thick dense mist or cloud, which completely obscures vision.

"Different opinions have been entertained regarding the cause of these bodies. Mr. Travers considers the fixed muscæ to be caused by a deposition of organized lymph, between the choroid and retina, compressing the papillæ of the retina, and the floating as merely a functional affection. Morgagni does not hesitate to express a belief that muscæ volitantes may arise from some injury done to the cells of the vitreous humour, from lesions of the optic nerve or retina, or from dilatation of the vessels which ramify through this nervous expansion. The pathology of muscæ volitantes does not appear, however, to be as yet known.

"Little silvery mots, together with a troublesome impression of the object looked at, remaining for some moments on the retina, are very common, and at the same time troublesome symptoms of amaurosis. These luminous sparks are most frequent during night, or when the eyes are shut, and are, as far as my observation goes, very unfavourable symptoms.

"Pain, to a certain extent, is almost always a constant attendant on amaurosis. Its seat and nature varies, however, and tends in many cases to throw some light on the nature of the exciting cause: thus, an internal hemicrania with vertigo enables us to recognise amaurosis, as probably dependant on plethora. The pain may be deep-seated in the eye-ball, with a constant feeling of fulness and weight, or it may be in the forehead and temple, and of a sharp darting nature. It may also be constant or fixed, remittent or periodical, and in other cases no pain whatever exists. In a patient affected with incomplete amaurosis, who saw objects of different colours, and was troubled with muscæ volitantes, a severe attack of headach came on regularly every afternoon; while he was perfectly free of pain during the remainder of the day."

II. The phenomena observed by the surgeon are of a much more positive nature. In many cases, indeed a single glance will detect the disease.

"A vacant stare of the eyes, with a rolling motion of the eyeballs, and inability to fix them steadily on any object, together with a degree of listlessness, or want of that animation so peculiarly characteristic of the healthy eye in many cases assures us of the existence of this affection. Besides visible organic alterations may exist, either as regards the colour or structure of the eyeball. These changes, however, in the colour, structure, or form of the eyeball, it is not my intention at present to consider. Nevertheless it may be stated, that they may consist in diseases of the cornea, of the pupil and anterior chamber, or in opacity of the tunica vitrea and chrystalline lens. The eyeball, too, is sometimes a degree less than the sound one, being apparently sunk in the orbit, and when pressed on with the fingers feeling as soft and yielding as a sponge.

"A change in the colour of the iris of the affected eye, is by no means unusual in this disease. The appearance which this membrane assumes, is regulated by its former colour, and will be more perspicuous when contrasted with the sound eye. This change in the colour of the iris, is always, as far as I have been able to observe, a mark of congestion, or deep-seated inflammation, more particularly if accompanied with a varicose state of the external vessels."

In a few rare cases our author has observed the anterior aspect of the iris become concave—for which it is very difficult to account. The iris becomes sometimes convex—a state, however, much more unfrequent than the former. An oscillatory motion of the iris is a very unfavourable symptom. Mr. Guthrie regards this vacillating motion, when combined with softness of the eyeball to the touch, as diagnostic of disorganization in the vitreous humour.

"Much information is to be derived in this disease from a careful examination of the state of the pupil. In the healthy eye, the pupil is always circular, and situated nearly in the centre of the iris. Its form and position varies much, however, in amaurosis. In some rare cases, as will afterwards be seen, it becomes perpendicularly oval, resembling the irides of the feline tribe; in others oblong; and even when it retains its natural form, it may be altered in position, being situated either higher or lower in the iris, to its nasal or temporal half. The occurrence of the perpendicular or oval pupil, is always to be regarded as a very unfavourable symptom.

"An active, and lively pupil, readily obeying the different impressions of light, has been regarded as a favourable symptom in amaurosis, more especially if this takes place when the sound eye has been covered, shewing that its movements are not from associated action; whilst a dilated and sluggish pupil little sensible to variations in light, has been regarded as very unfavourable. The degree of information, however, to be derived from the motions of the pupil, is rather of a limited kind, and not to be relied on implicitly. As in some cases of complete amaurosis, the pupil may be active and lively; and a dilated sluggish pupil may be found in others where no amaurosis exists. The pupil will be seen to have been active and lively, in the fourth case, to be afterwards related, although the amaurosis was complete. On the other hand, the pupil may be immoderately dilated, in consequence of adhesions formed between the posterior surface of the iris, and capsule of the lens, the usual result of iritis or deep-seated inflammation.

"In amaurosis the transparent parts of the eye, are subjected to opacities of different degrees, and kinds. Vision may be much diminished by depositions of lymph on the delicate structure of the retina, or on the surface of the lens; these, however, as they cannot be seen, do not fall to be considered here.

"A deep-seated opacity, of a pale yellowish green colour and shaded off towards its circumference, is one of the most frequent changes of the transparent parts of the eye in amaurosis. Considerable difference exists with regard to the intensity of this opacity; the humours being at one time perfectly green, and at another only observably opaque after a minute examination. In its appearance it is not unlike the cataract of old people, and from its situation may very readily be mistaken for such by the inexperienced. Mr. Travers has known the operation of extraction performed, in a case where this opacity was mistaken for cataract. It is said to have its seat in the vitreous humour, or its capsule, and is always to be regarded as a very unfavourable symptom ; although the same greenish appearance is frequently to be seen in the eyes of many old people in whom no defect of vision exists, farther than is to be met with at their advanced age.

"A greyish milky opacity is also sometimes met with in such cases, situated immediately behind the pupil, and which is much more readily mistaken for cataract. However, its comparative thinness, and depth behind the pupil, will in most cases serve to discriminate between them."

The causes of amaurosis are very numerous, and very opposite in their nature. Mr. Travers has divided them into functional and organic, and this classification Mr. Knox adopts.

"*Species 1st. Amaurosis from atony of the nerves of the eye.* Suppressed, diminished, or abolished vision may be the result of atony of the nerves of the eye, dependant on general atony of the constitution. Whatever has a tendency to weaken the system generally, must operate in like manner on the nerves of the eye ; hence it is no uncommon circumstance to find amaurotic affections arising from those general debilitating causes. Under this head a number of exciting causes may be enumerated ; such as excessive discharges of any kind, as leucorrhœa, too frequent indulgence in venery, long protracted lactation ; intemperance, and probably typhus. I have only once met with a case of amaurosis, arising, as I thought, from long protracted lactation. The patient was a middle-aged woman, of a spare habit of body, and mother of a large family. She was almost perfectly blind. On examining her eyes, no morbid appearance was visible. She had been nursing nearly fourteen months, and was but poorly fed. A variety of means were tried, but without effect. A medical friend of mine informs me, that a lady, a patient of his, and the mother of a large family, invariably towards the termination of lactation, becomes troubled with amaurotic symptoms. The same gentleman also informs me of a case having occurred lately in a wet nurse. Instances are on record of females being amaurotic during pregnancy, and recovering their sight after delivery. Those who are anxious of meeting with such cases, may consult Rolfino.

"The general habit of body, of a person labouring under this species of amaurosis, is leuco-phlegmatic. There is great lassitude and debility. On examining the eyes, the pupils are found to be dilated, and very sluggish. No organic change is visible in the interior of the eye, a circumstance denoting the purely functional nature of the affection. There is also a troublesome sensation of black motes, or stars before the eye, rarely any flashes of fire, and the pain when present is but trifling. . . .

"Case 1. *Amaurosis from Debility and Leucorrhœa.*—Mrs. ——, on application, complained of a diminution of vision. Pupils were enlarged and sluggish, but jet black ; had occasional pain in the eye ; complained much of general debility, and leucorrhœa. This patient was in a short time relieved by the use of the tincture of iron, and vapour of ammonia.

"Case 2. *Amaurosis from Intemperance.* William Frazer, æt. 45, has all his life been subject to idiopathic weakness of sight ; is a dram-drinker. A week previous to application, he lost the sight of the right eye, in the course of a day. A troublesome sensation of black stars, to use his own words, and of black objects, first warned him of its state. At present, he can discern nothing, not even strong light ; pupil the same as the left one ; both, however, are almost insensible to variations of light, but obey the belladonna ; has no headach, or apparent plethora of head ; has all his life had but a poor appetite, and weak digestion. Believes this attack to have been brought on, or hastened by getting tipsy three days before application. He was ordered a blue pill morning and evening, and a sternutatory composed of sulphate of hydrarg. et pulv. glycyrrhizæ, every night at bedtime. On the following morning, he stated that, during the action of the snuff, he perceived his sight to return in some degree. Half an ounce of blood was taken from the angular vein, and two drops of vin. opii prescribed to be put into the eye every night. Four days after application, vision was much improved. The following embrocation to be rubbed on the forehead and temples every night. ℞ Alcohol. ℥i. aq. ammon. ʒij. M. Has continued the use of the pills, embrocation, drops, and sternutatory, and can now see as well with the right as with the left eye; but observes, sometimes in the evening, that its power of vision is suddenly obscured. Ten days after application, he was dismissed well."

TREATMENT.

This is too often empirical—hence bleeding, blistering, mercury, &c. have been recklessly and indiscriminately employed, without ascertaining the etiology of the complaint—the first thing that ought to be done.

"The treatment of amaurosis from debility consists in removing the supposed cause, and in strengthening the system, by the exhibition of tonics and diffusible stimuli.

"To effect this, the whole class of medicines denominated tonics may be used with advantage; but the sulphate of quinine, and the preparations of iron, are most to be relied on. The carbonate, and tincture of the muriate, of iron are the preparations of that metal that will be found most advantageous.

"Diffusible stimulants, by imparting a temporary excitement to the retina, are often of great service. For this purpose, a sternutatory composed of the sulph. hyd. flav. and pulv. glycyrrhizæ, in the proportion of a scruple of the former to an ounce of the latter, answers the purpose well. I have often seen very great advantage derived from their use. The vapour of ammonia received on the eyeballs is also worthy of a trial. An embrocation composed of alcohol and ammonia, in the proportions used in case second, may be rubbed morning and evening on the forehead and temples with advantage.

"Bloodletting, and the use of mercury, are, in most cases of amaurosis dependent on debility, injurious. Blisters are sometimes useful, and may be applied behind the ears, on the temples, and on the forehead, but they ought not to be kept open, as every drain on the constitution is hurtful.

"*Species 2d. Amaurosis from Plethora.* Amaurosis may arise from a state of the system the very reverse of the above, viz. from plethora. When amaurosis depends on a plethoric state of the system, the symptoms are very unequivocal. There is a florid complexion, with more or less tumefaction of the face; vertigo, with a feeling of fulness and weight in the eyeball and head; the pupil is also sluggish and dilated. All those causes which have a tendency to increase the activity of the system, powerfully predisposes to this species of amaurosis. Among them may be ranked high living, and perhaps the sudden suppression of any accustomed discharge, as epistaxis, hæmorrhoids, or the catamenia. In the case of Turnbull, afterwards to be related, it will be seen that for many years previous to the attack, he had been subjected to hæmorrhoids, and that, on their cessation, amaurosis supervened.

"A temporary determination, or congestion of blood, may also take place in the eye and brain of patients who are not otherwise of a full habit of body, and thus give rise to amaurotic symptoms. I have lately observed this in a patient under my care, with disease of the heart, who became alarmed at finding the sight of one of the eyes failing, and, at the same time, saw objects double and very indistinctly. When the temporary irritation ceased, the balance of the circulation was restored, and the amaurotic symptoms disappeared.

"The effects produced on the retina by congestion, or determination of blood, are probably pressure on its delicate structure. It is difficult to draw the line of distinction between inflammation and congestion; and it is, therefore, not improbable, that some of the products of that process, such as thickening and lymphatic deposits, may be found on the retina. This opinion is strengthened by the fact, that, even after the determination has been relieved, amaurotic symptoms continue, until those means calculated to remove depositions of lymph have taken effect. The central artery of the optic nerve becoming distended with blood, is also capable of producing a powerful pressure on its substance; and, besides, when it is remembered that the choroid coat, immediately exterior to the retina, is composed entirely of blood-vessels, it is easy to imagine how its delicate structure may be compressed against the vitreous humour, and thus paralyzed, when its vessels become turgid with blood. That this actually does take place, is clearly proved by the signal relief experienced when blood is abstracted in such a case.

"Case 3. *Amaurosis from Suppressed Catamenia.* A young robust woman applied on account of a diminution of her vision. Saw objects dimly, and covered with mots; pupils were enlarged, and sluggish, but jet black; eyebrows, also, were swollen, and felt heavy. Stated that the disease came on a month previous to application, after a sudden suppression of the catamenia. An emetic with emmenagogue medicines were prescribed. Three days after application, a great change to the better; saw objects clearer and free from mots; eyelids, however, still appear swollen, in consequence of which she was bled to twelve ounces, and in a few days dismissed cured.

"Case 4. *Amaurosis from Plethora, with Moveable Pupil.* Mr. —— applied on account of nearly a total loss of sight in the left eye, and diminution of it in the right. He lost the sight of the left eye a year before application. The iris was of a deep green colour, and the pupil readily obeyed the different impressions of light. After the application of the belladonna, the humours were observed to be slightly muddy. He could not distinguish the outlines of large objects with the left, and, with the right, was only sensible of the difference between light and darkness. The eyeball was of its natural size, and firm to the touch; the other membranes were natural in appearance. At the time he lost the sight of the left eye, he had no pain or uneasiness in it. A variety of remedies were adopted in this case without the smallest benefit. They were the following:—Repeated blood-letting and blistering, a course of mercury of four weeks' duration, and the application of the opiate wine to the eyes.

"*Treatment.* The treatment of this species of amaurosis consists in adopting all those means which are calculated to lower the tone of the system. Hence the abstraction of blood both local and general, together with abstinence, are chiefly to be relied on. The quantity of blood to be taken must be regulated according to the constitution of the patient, and repeated as often as necessary.

"It sometimes, however, happens that when the balance of the circulation is lost, that abstraction of blood produces only a temporary relief. I once met with a case of this kind. The patient was a strong athletic man from Ayrshire, who applied to me, in February, 1828, in consequence of a loss of vision. This patient bore evident marks of plethora; the face was full and florid; pupils enlarged, and sluggish; the external tunics were also varicose, together with a vascular zone between the sclerotic and cornea. He was cupped on both temples, and put on a course of mercury. For a few days, vision was much improved, but symptoms of congestion returned, and it became as bad as ever. The same thing happened again and again in despite of repeated bloodlettings, and at last he was dismissed uncured.

"Next to the abstraction of blood, the exhibition of mercury operates most powerfully in arresting the progress of inflammation. This is most beautifully seen in iritis, when, as soon as that mineral has taken effect, a decided check is given to the inflammation, and the already effused lymph is absorbed. The same thing happens in congestion, or inflammation of the deep-seated parts of the eye. The bowels must also be kept freely open, by means of saline purgatives combined with antimonials. Blisters should be frequently applied, and a constant discharge maintained, by means of an issue in the nape of the neck. The diet must also be strictly antiphlogistic. When there is reason to suppose the attack brought on by the sudden disappearance of any accustomed discharge, medicines, calculated to re-establish it, should be administered. The hair on the head should also be kept short.

" *Species 3d. Amaurosis from Sympathy.* Amaurosis is also sometimes produced from the retina sympathizing with some other part of the body. This species might with propriety be called amaurosis sympathetica, vel gastrica.

"The remote sympathies that exist between the different parts of the body, are often truly astonishing, and with none are these more strikingly exemplified, than with the abdominal viscera. Hence amaurotic affections are often produced by gastric impurities, and derangements of the primæ viæ. Many examples are on record of amaurosis being produced by a disordered state of the alimentary canal, as well as after eating a hearty meal. The most marked example, however, of amaurosis being produced from sympathy, is that which is often met with in children troubled with worms. I have only once met with a decided case of this kind. The patient, a child seven years of age, was brought to me in August 1828. The case is entered in my eye case book as corneitis of left eye, with sympathetic amaurosis of both from worms. Small doses of calomel and scammony were prescribed with the happiest effect, a great number of ascarides being discharged. In proportion as these were removed the amaurotic symptoms disappeared. The following in general will be found to be the symptoms, when amaurosis is produced by worms. Eyeballs natural in appearance, pupils very much dilated and sluggish, but jet black, belly generally tumid, or enlarged, appetite voracious, with picking at the nose.

"One of the most remarkable, and at the same time distressing cases of amaurosis from sympathy that I have heard of, occurred lately. The patient, a young lady, had a needle in the ribbon around her waist, which accidentally run into the hand near the wrist, and broke. The family surgeon had to be sent for to remove it. Severe pain was experienced at the time, and continued for some time afterwards. She became on the following day (I believe) amaurotic of one eye, and of the other also in a few days after.

"This and the first species of amaurosis are the only instances, as far as I have observed, that are ever remittent, intermittent, or periodical. For those cases which depend on the greater clearness of one day, or part of it, over that of any other, are not to be considered either as remittent, or periodical, as they obviously depend on the degree of light presented to the eye, and not on any variation of the powers of vision. They also admit of being oftener cured than any of the other species, because they do not depend on any organic change of the eye, or its appendages, and hence it is that the pupil in them is always jet black.

" *Treatment.* It is in this species of amaurosis that the emeto-purgative plan of Scarpa and Richter will be found most serviceable. If, as sometimes happens, the amaurosis should have succeeded a hearty meal, and there is reason to suppose that the disease is produced by it, an emetic should be administered, and repeated at proper intervals. The bowels ought also to be kept freely open, with pills composed of calomel and colocynth, or any other strong purgative.

"When worms are the supposed cause of the affection, medicines calculated to remove these should be administered. A combination of calomel and scammony I have found a most valuable anthelmintic. Turpentine enemata are also highly beneficial.

"The treatment of sympathetic amaurosis from wounds, must be regulated by existing symptoms. The free division of the part as recommended by Beer and others in amaurosis consequent on wounds of the superciliary nerves should not be lost sight of, as in some cases of the above description, it has been found serviceable. Strict attention must also be paid to the state of the alimentary canal."

We shall be very glad to see the second part of Mr. Knox's paper, and hope to profit by its perusal.

